# CO_2_ Sensing Characteristics of a La_2_O_3_/SnO_2_ Stacked Structure with Micromachined Hotplates

**DOI:** 10.3390/s17092156

**Published:** 2017-09-20

**Authors:** Tatsuya Iwata, Kyosuke Matsuda, Kazuhiro Takahashi, Kazuaki Sawada

**Affiliations:** Department of Electrical and Electronic Information Engineering, Toyohashi University of Technology, Toyohashi 4418122, Japan; matsuda-k@int.ee.tut.ac.jp (K.M.); takahashi@ee.tut.ac.jp (K.T.); sawada@ee.tut.ac.jp (K.S.)

**Keywords:** CO_2_ sensor, micromachined hotplate, La_2_O_3_, SnO_2_

## Abstract

Demand for the detection of carbon dioxide (CO${}_{2}$) is increasing in various fields, including air-quality monitoring, healthcare, and agriculture. On the other hand, smart gas sensors, in which micromachined gas sensors are integrated with driving circuits, are desirable toward the development of the society of the internet of things. In this study, micromachined hotplate-based CO${}_{2}$ sensors were fabricated and their characteristics were investigated. The sensors have La${}_{2}$O${}_{3}$/SnO${}_{2}$ stacked layers as a sensing material and Pt interdigitated electrodes. A CO${}_{2}$ response of 2.9 for a CO${}_{2}$ concentration of 1000 ppm was obtained at 350 °C with low power consumption (approximately 17 mW). A relatively large response was obtained compared with previous studies even though a compact sputtered-SnO${}_{2}$ film was used. This high response was speculated to be due to a significant contribution of the resistance component near the electrode. Furthermore, CO${}_{2}$ sensing was successfully performed in the CO${}_{2}$ range of 200–4000 ppm with at least 200-ppm resolution.

## 1. Introduction

Carbon dioxide (CO${}_{2}$) exists in air at a fraction of approximately 400 ppm, and its detection is highly demanded for air-quality monitoring, healthcare, and agriculture. On the contrary, with the development of the internet of things and smart sensors, which have functions of signal processing and communication besides sensing, have attracted much attention. Furthermore, sensor chips, into which these functions are integrated, are favorable for the miniaturization of such smart sensors [1,2,3].

Till date, non-dispersive infrared absorption (NDIR) type, which shows a wide measurement range and high accuracy (e.g., 0–5000 ppm with ±30 ppm accuracy for K30, Sensair [4]), is most widely used as CO${}_{2}$ sensors. However, it is difficult to integrate a NDIR sensor into a chip due to the necessity of a bulky optical system. On the other hand, electro-mechanical [5], potentiometric [6,7,8], and conductometric types [9,10,11,12,13,14,15,16,17] have been proposed as miniature CO${}_{2}$ sensors, feasibly integrated into a chip. In particular, the conductometric-type is most suitable for smart sensors in terms of simplicity of readout circuits and IC-process compatibility. Table 1 compares the properties of various conductometric sensors. La-compound-based sensors show a relatively large response, while their operation temperature is several hundred °C. On the contraty, carbon nanotube [16], graphen oxide [15], and poly(ethyleneimine) (PEI) functionalized-polyaniline (PANI) [17] operate at room temperature (RT), their responses are relatively low. Taking account of the application to smart sensors, in which signal processing is carried out on a chip, a large response is beneficial to obtain a sufficient signal–noise ratio of the output signal. In this regard, La-compound materials are promising for smart CO${}_{2}$ sensors.

As described above, La-compound materials require heating to several hundred °C for sensing CO${}_{2}$, resulting in large power consumption (typically several hundred mW) and a rise in the temperature of the sensor substrates. Therefore, a micromachined hotplate (MHP), which is a suspended heating structure based on microelectromechanical systems, is employed to thermally isolate the sensors from the surroundings [18]. By employing MHPs, the power consumption for the heating is reduced by more than one order of magnitude. In addition, the temperature rise in the surrounding area of the hotplate is suppressed, and thus, the integration of electronic circuits is also possible [2,19,20]. MHP-based semiconductor gas sensors were reported in the sensing of hydrogen (H${}_{2}$) [21], carbon monoxide (CO) [2,22,23], and nitrogen oxide (NO${}_{x}$) [3,24], however, semiconductor CO${}_{2}$ sensors based on MHPs have not been demonstrated.

On the other hand, the response of semiconductor gas sensors can change with sensor size and the material and structure of electrodes besides the type of materials and their microstructures [25,26,27]. In addition, although La-based semiconductor sensors show large responses to CO${}_{2}$ as described above, the responses to CO${}_{2}$ are still low due to the chemical inertness of CO${}_{2}$ when compared with those for reactive gases such as CO and H${}_{2}$. For example, to our best knowledge, LaOCl-functionalized SnO${}_{2}$ nanowires (SnO${}_{2}$-NW) [14] showed the highest response of 4.5 for 1000-ppm CO${}_{2}$ amongst the semiconductor materials for CO${}_{2}$ detection, as shown in Table 1. Furthermore, its sensor area was 800μm×1600μm, and, thus, a structure with a smaller area and a MHP is required for smart sensors with low power consumption. Taking account of these relatively low responses of CO${}_{2}$ sensors, the influence of the sensor structure on the response can be a critical issue when the sensor size is to be shrunk. Therefore, CO${}_{2}$ sensors fabricated on MHPs should be characterized toward the realization of smart CO${}_{2}$ sensors. In this study, sensor elements with La${}_{2}$O${}_{3}$/SnO${}_{2}$ stacked layers as sensing materials were fabricated on MHPs, and their sensing characteristics were investigated.

## 2. Experimental Procedure

### 2.1. Sensor Fabrication

**Figure 1:**
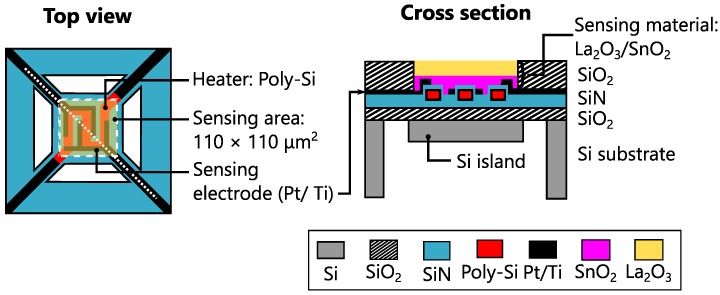
Schematic illustration of the device. The left and right figures represent its top and cross-sectional views, respectively. The location of the cross section is indicated as a white dotted line in the top view. Note that the upper SiO${}_{2}$ layer is not shown in the top view for the ease of understanding.

The sensors were fabricated on MHPs. A schematic illustration of the devices is shown in Figure 1, which depicts both top and cross-sectional views, and the white dotted line in the top view indicates the position of the cross section. The MHP has a membrane comprising of stacked layers of silicon dioxide (SiO${}_{2}$) and silicon nitride (SiN), suspended with four bridges. The heater has a meander-shaped structure, and polycrystalline Si (poly-Si) is used as the heater material. Under the membrane, an approximately 50-μm-thick Si island was formed to maintain a uniform temperature distribution within the membrane. The sensor part comprises the sensing layer of an La${}_{2}$O${}_{3}$/SnO${}_{2}$ stack and platinum interdigitated electrodes. The electrodes have a width of 10 μm and a spacing of 20 μm.

First, SiO${}_{2}$ with a thickness of approximately 620 nm was thermally grown on a Si substrate. A 100-nm-thick SiN was then deposited by low-pressure chemical vapor deposition (LPCVD). Poly-Si with a thickness of approximately 330 nm was deposited on the SiN/SiO${}_{2}$ layer by LPCVD. The phosphorus doping was carried out by thermal diffusion using phosphorus oxychloride gas at 1050 °C. The sheet resistance of the doped poly-Si was approximately 17 Ω/sq, which corresponded to the resistivity of 5.6 × 10${}^{-4}$Ω cm. Poly-Si was then patterned by reactive ion etching to form the heater resistor. A SiN layer with a thickness of 100 nm was deposited by LPCVD, as an insulation layer between the heater and the sensor. A Pt/Ti layer was then deposited by sputtering and patterned by a lift-off process to form the electrode. The Pt/Ti layer was also used as a wiring of the heater. As a protective layer of the wiring, an SiO${}_{2}$ layer was deposited by plasma-enhanced CVD. The thickness of the SiO${}_{2}$ layer was approximately 500 nm. After exposing the sensing electrodes by etching the SiO${}_{2}$ layer to define the sensing area, SnO${}_{2}$ was deposited by sputtering, and its thickness was approximately 100 nm. For La${}_{2}$O${}_{3}$ deposition, metal-organic decomposition material (La-03, Kojundo Chemical Lab. Co., Sakado, Japan) was used as a precursor and was spin-coated on the sample with a rotational speed of 4000 rpm. Then, the sample was prebaked at 200 °C in the air for 30 min, followed by rapid thermal annealing (RTA) at 650 °C in an O${}_{2}$ atmosphere for 70 s. The area of the sensing material was 110 μm × 110 μm, which was patterned by a liftoff process. Finally, the membrane was released by deep-reactive ion etching followed by xenon difluoride etching from the backside. The membrane area was 140 μm × 140 μm, and the effective area of the device including the bridges was approximately 270 μm × 270 μm. The fabrication details of the MHP are described elsewhere [28].

### 2.2. Measurement Procedure

The structure of the sensing layer was observed by scanning electron microscopy (SEM) with an acceleration voltage of 10 kV. The observation was conducted on the sensor elements fabricated on SiO${}_{2}$/Si substrates (not on the MHP).

**Figure 2:**
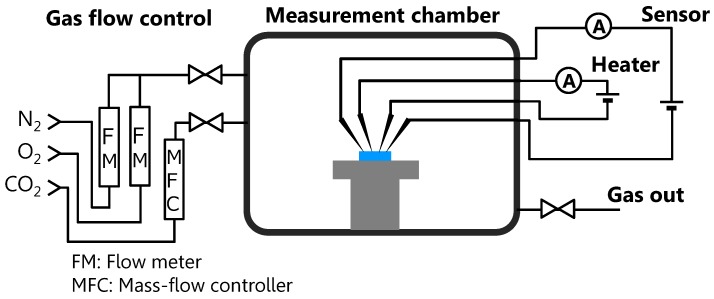
Schematic illustration of the measurement system. Three gas lines (N${}_{2}$, O${}_{2}$, and CO${}_{2}$) are equipped with the system, and these gases are introduced into the chamber through flow meters and a mass–flow controller.

Electrical measurements were performed in a chamber with a gas-control system, as shown in Figure 2. The chamber has two gas inlets and one outlet, and the atmosphere in the chamber was controlled by changing the flow rate of the used gases. The system has three gas lines (N${}_{2}$, O${}_{2}$, and CO${}_{2}$), and these gas flows were controlled by flow meters for N${}_{2}$ and O${}_{2}$, and a mass-flow controller for CO${}_{2}$. In the chamber, the current flowing through the sensing elements was measured by applying a bias voltage of 0.5 V. During the measurements, a mixture of N${}_{2}$ and O${}_{2}$, as synthesized air, was introduced into the chamber with a constant flow rate (N${}_{2}$: 1.6 slm and O${}_{2}$: 0.4 slm). Then, CO${}_{2}$ was introduced into the chamber to investigate the CO${}_{2}$ response at various CO${}_{2}$ concentrations and heater temperatures, at which the CO${}_{2}$ concentration was set by controlling the flow rate of CO${}_{2}$. The CO${}_{2}$ response was defined as the quotient of the resistance in the atmosphere without CO${}_{2}$ ($R_{a}$) and with CO${}_{2}$ ($R_{{\mathrm{CO}}_{2}}$), $R_{a}/R_{{\mathrm{CO}}_{2}}$.

## 3. Results and Discussion

### 3.1. Observation of the Sensing Layer

**Figure 3:**
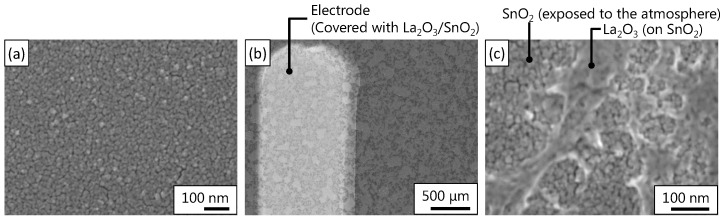
Scanning electron microscopy images of the material: (**a**) SnO${}_{2}$ surface; (**b**) La${}_{2}$O${}_{3}$/SnO${}_{2}$ surface; and (**c**) the magnified image of (**b**).

Figure 3a shows an SEM image of the SnO${}_{2}$ layer. The SnO${}_{2}$ layer has a compact structure with a grain size range of 20–40 nm. Figure 3b,c depict the images of the La${}_{2}$O${}_{3}$/SnO${}_{2}$ layer at different magnifications. The images (b) and (c) reveal that the La${}_{2}$O${}_{3}$ layer was discontinuously deposited on the SnO${}_{2}$ layer, which was partly exposed to the atmosphere. In addition, the grain structure was not clear in the La${}_{2}$O${}_{3}$ layer. The discontinuous layer of La${}_{2}$O${}_{3}$ may have resulted from the deposition condition including a spin-coating, a lift-off process, and RTA. Given that CO${}_{2}$ responses change with the concentration of La${}_{2}$O${}_{3}$ in the mixture of La${}_{2}$O${}_{3}$ and SnO${}_{2}$ [10], the coverage of the La${}_{2}$O${}_{3}$ layer possibly influences the response to CO${}_{2}$ and the selectivity, and thus, the deposition condition of La${}_{2}$O${}_{3}$ layer should be investigated. Nevertheless, the situation that both the SnO${}_{2}$ and La${}_{2}$O${}_{3}$ layer were exposed to the atmosphere is similar to previous studies [10,14]. Thus, sensitization by the La${}_{2}$O${}_{3}$ layer should be obtained in this regard.

### 3.2. Characteristics of Micromachined Hotplates

**Figure 4:**
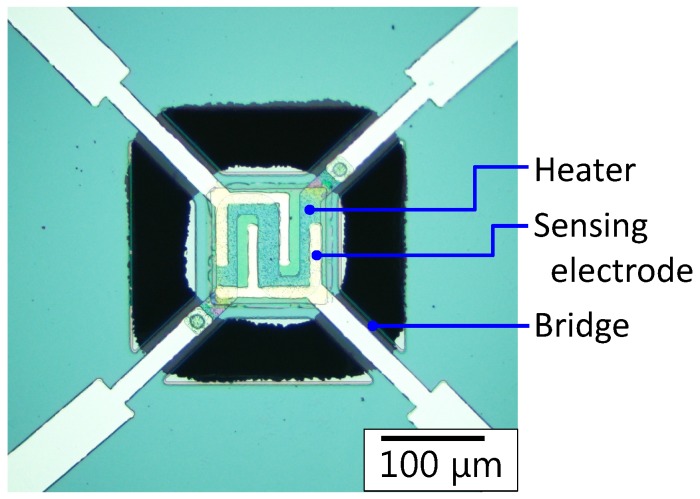
Optical microscopy image of the fabricated device.

**Figure 5:**
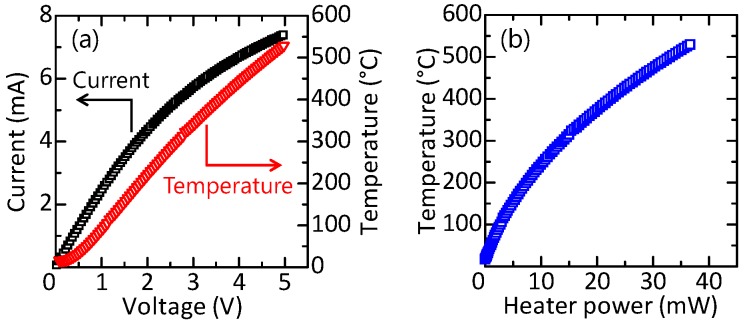
(**a**) I−V characteristics of the heater. The corresponding temperature calculated from the heater resistance is also plotted; (**b**) The temperature of the heater plotted against the power consumption.

As the optical microscope image in Figure 4 shows, the device was successfully fabricated. Then, the heater of the MHP was characterized. Figure 5a shows the current–voltage (I−V) characteristics of the heater. In addition, the heater temperature calculated from the heater resistance [28] was plotted on the graph. It was confirmed that the MHP can be heated to approximately 530 °C at 5 V. The heater temperature plotted against the power consumption of the heater is shown in Figure 5b. The characteristics show that the power consumption at 400 °C, which is a typical operating temperature of semiconductor CO${}_{2}$ sensors, was approximately 23 mW.

### 3.3. Temperature Dependence of the CO${}_{2}$-Sensing Characteristics

**Figure 6:**
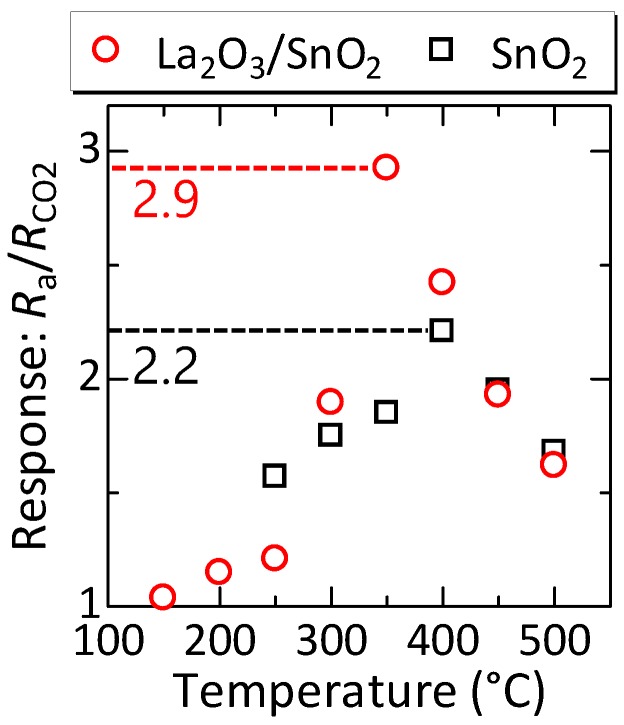
Temperature dependence of the CO${}_{2}$ response of the sensors. The sensitivities of both SnO${}_{2}$ and La${}_{2}$O${}_{3}$/SnO${}_{2}$ elements are plotted. SnO${}_{2}$ showed the highest response of 2.2 near 400 °C, whereas La${}_{2}$O${}_{3}$/SnO${}_{2}$ showed a higher response of 2.9 than SnO${}_{2}$ near 350 °C.

The response for 1000-ppm CO${}_{2}$ obtained from the resistance–time (R−t) characteristics at different temperatures is shown in Figure 6. The result for the SnO${}_{2}$ element is also shown for comparison. In both elements, the response increased as the temperature increased, reached a peak value at a certain temperature, and then decreased with further increasing temperature. The response of the La${}_{2}$O${}_{3}$/SnO${}_{2}$ element was smaller than that of SnO${}_{2}$ at low temperatures (e.g., near 250 °C), while it exceeds that of the SnO${}_{2}$ element above 250 °C. The maximum response of approximately 2.9 was obtained near 350 °C for the La${}_{2}$O${}_{3}$/SnO${}_{2}$ element, whereas the SnO${}_{2}$ element exhibited a maximum response of 2.2 near 400 °C. Above 450 °C, the response of both elements decreased with increasing temperature, and they exhibited similar responses. As a result, a higher maximum response was obtained for the La${}_{2}$O${}_{3}$/SnO${}_{2}$ element, namely, sensitization by La${}_{2}$O${}_{3}$ was obtained in the device on the MHP. The power consumption at 350 °C of the MHP was approximately 17 mW, which is smaller by approximately one order of magnitude than that of the conventional semiconductor sensors [29].

For the SnO${}_{2}$ element, it is reasonable to ascribe the CO${}_{2}$ response to the chemical adsorption (chemisorption) of CO${}_{2}$ on the surface. In contrast, given that the sensitization by the deposition of La${}_{2}$O${}_{3}$ was observed, the chemisorption of CO${}_{2}$ on the La${}_{2}$O${}_{3}$ plays an important role in the response of the La${}_{2}$O${}_{3}$/SnO${}_{2}$ element. It was suggested that carbonate formation by the reaction between La${}_{2}$O${}_{3}$ and CO${}_{2}$ is the main cause of the CO${}_{2}$ response [11,30], and the suggested reactions are as follows [30].

(1) $$\begin{array}{cc} O^{2-}+{\mathrm{CO}}_{2} & \to {\mathrm{CO}}_{3}^{2-} \end{array}$$

(2) $$\begin{array}{cc} {\mathrm{CO}}_{2}+{\mathrm{CO}}_{3}^{2-} & \to C_{2}O_{4}+O^{2-} \end{array}$$

On the other hand, because the La${}_{2}$O${}_{3}$ layer is discontinuous and La${}_{2}$O${}_{3}$ generally exhibits much larger resistivity than SnO${}_{2}$, almost all the current should flow through the SnO${}_{2}$ layer, while La${}_{2}$O${}_{3}$ acts as a sensitization layer in this study. Thus, carrier density in the SnO${}_{2}$ layer must be modulated accompanying to the above reactions, whereas these reactions do not directly cause carrier transfer. Although the detailed mechanism of the carrier transfer is not clear and should be further investigated, one possible scenario is the trap/detrap of carriers from the states at the interface between SnO${}_{2}$ and La${}_{2}$O${}_{3}$. It is likely that the work function of La${}_{2}$O${}_{3}$ is changed by the CO${}_{2}$ chemisorption at the surface, and this work function change might be related to the trap/detrap of carriers.

Here, the temperature dependence of the response is discussed. These chemisorption is generally a thermally activated process, competing with physical adsorption (physisorption), and, therefore, as temperature increased, the probability of chemisoption increases [31]. On the contrary, by further increasing the temperature, desorption of the molecule may become dominant, resulting in the response being decreased at higher temperature. The activation energy for chemisorption and desorption of CO${}_{2}$ depends on the adsorbate, causing a difference in the temperature dependence of the response between SnO${}_{2}$ and La${}_{2}$O${}_{3}$/SnO${}_{2}$. CO${}_{2}$ chemisorption on SnO${}_{2}$ can occur at low temperatures [32], and the two kinds of activation energy for the desorption were reported as 0.18 and 0.06 eV. The energy for chemisorption should be smaller than those energies. On the other hand, Esaka et al. conducted differential thermal analysis (DTA), and reported that the significant adsorption of CO${}_{2}$ on Bi${}_{2}$O${}_{3}$–La${}_{2}$O${}_{3}$ under CO${}_{2}$ atmosphere occurred near 400 °C [33]. Bakiz et al. reported that the transformation of La${}_{2}$O${}_{3}$ to La${}_{2}$O${}_{2}$CO${}_{3}$ (carbonatation) occurred near 520 °C [34], and the activation energy for the carbonatation at the oxide surface to be 7.6 eV. Although these specific temperatures, which depend on the material and experimental conditions, are just for reference, it is likely that the CO${}_{2}$ chemisorption on La${}_{2}$O${}_{3}$ has larger activation energy than that on SnO${}_{2}$. Therefore, the probability of CO${}_{2}$ chemisorption on La${}_{2}$O${}_{3}$ may be small at low temperatures, and the response to CO${}_{2}$ at low temperatures may be larger in the SnO${}_{2}$ than in La${}_{2}$O${}_{3}$/SnO${}_{2}$.

Concerning the response decrease at high temperatures, Marsal et al. reported that the CO${}_{2}$ desorption from LaOCl was observed in the temperature range of 260–380 °C by TPD analysis [30]. Given that the desorption temperature depends on the material and microscopic surface structure, as just mentioned, the temperature at which the CO${}_{2}$ response began to decrease in this study (above 350 °C) roughly corresponds to such a desorption temperature. As a result, the response may reach the maximum near 350 °C in this study. Similar temperature dependencies, in which the response reached a maximum at a certain temperatures, were also reported in previous studies on La-compound-based CO${}_{2}$ sensors [14,35]. In contrast, for SnO${}_{2}$, Dobrovolsky et al. reported that the thermal desorption spectrum of CO${}_{2}$ adsorbed on SnO${}_{2}$ at 20 °C exhibited a desorption peak near 400 °C. Although the temperature for the maximum response of the SnO${}_{2}$ element is speculated to be lower than that of La${}_{2}$O${}_{3}$/SnO${}_{2}$ because of the smaller activation energy for CO${}_{2}$ desorption from SnO${}_{2}$ than that from La${}_{2}$O${}_{3}$ (activation energy for desorption is generally larger than that for chemisorption), taking account of the study in Ref. [32], it might be possible that the SnO${}_{2}$ element exhibited a maximum response to approximately 400 °C.

Table 2 compares the properties of the La-compound-based CO${}_{2}$ sensor with those in the previous studies. As shown in Table 2, the response of the sensor in this work is high, although it is not the highest. Further, our device operates with low power consumption owing to the MHP, whereas the others are not fabricated on MHPs. Namely, this work demonstrates a semiconductor CO${}_{2}$ sensor with both a fairly good response and low power consumption. Note that the MHP is heated to a driving temperature (several hundreds °C) with a short period (∼100 ms) due to its small thermal capacity. Thus, intermittently driving the sensor with short pulse inputs is possible, which enables a further decrease in the power consumption, as demonstrated in Ref. [36]. Furthermore, the MHP-based sensors are also beneficial to improve selectivity, which is one the most critical properties of gas sensors. The sensor in this study is likely to have poor selectivity to other reducing gases such as CO and H${}_{2}$ because the SnO${}_{2}$ layer was partly exposed to the air. For this issue, using a reference sensor and reading differential output between the sensor and the reference is one solution. However, using a reference sensor is rather problematic for conventional metal oxide sensors because it causes an increase in module size and the power consumption. In contrast, MHP-based sensors allow us to easily integrate multiple elements into a single chip, because they are fabricated by a monolithic IC process. Therefore, in conjunction with their capability of low-power operation, it is possible to improve the selectivity as a chip by realizing such an array in which a sensor and a reference are simultaneously integrated, while also keeping their advantages of miniature size and low power consumption.

Another interesting result is that the relatively high response was obtained for a sputtered-SnO${}_{2}$ film, which formed a compact layer, as shown in Figure 3. Generally, porous layers show higher response than compact layers due to their large surface–volume ratio for reducing gases such as CO and H${}_{2}$ [25,27]. Despite that, our device showed a relatively high response (see Table 2). We ascribe this response to the contribution of the resistance near the electrode contacts. In compact layers, the resistance component near the contact tends to become large as compared with that in porous films because the resistance at the grain boundaries can be small in compact layers. Thus far, several studies reported the contribution of the resistance near the contacts to the detection [37,38]. Hoefer et al. have shown that the resistance near the contacts significantly contributed to detection in sputtered films [37]. In a similar manner, the contribution of the resistance near the contacts may be significant in this study, thus, a higher response was obtained than in other studies in which porous thick films were used. In addition, it is indicative that the LaOCl-functionalized SnO${}_{2}$-NW showed a high response, in which the SnO${}_{2}$-NW was more than 10 μm long, and the electrode gap was 20 μm [14]. Because the length of the NW and the electrode gap were of the same order of magnitude, the number of the contacts between NWs become much less than the number of grain boundaries in porous layers. As a result, the component of the contact resistance can become large, which may contribute to the high response. Further investigation of the effect of the resistance near the contacts on CO${}_{2}$ detection is under way.

### 3.4. Concentration Dependence of the CO${}_{2}$ Response

**Figure 7:**
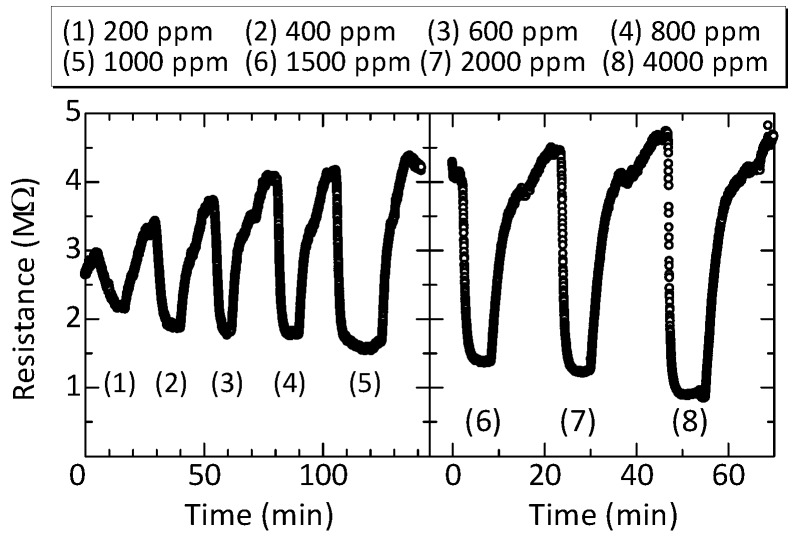
Time-dependent resistance change of the La${}_{2}$O${}_{3}$/SnO${}_{2}$ element for different CO${}_{2}$ concentrations. The measurement was interrupted between (5) and (6) for several tens of a second because of the limitation of the measuring instrument.

The response of the sensor for different CO${}_{2}$ concentrations was measured at 400 °C, at which the power consumption of the heater was approximately 23 mW. Figure 7 shows the time-dependent resistance change of the La${}_{2}$O${}_{3}$/SnO${}_{2}$ element when it was exposed to CO${}_{2}$ with different concentrations: (a) 200–1000 ppm and (b) 1500–4000 ppm. Note that the measurement was interrupted between (a) and (b) because of the limitation of the measuring instrument. Although a baseline resistance drift was observed, the magnitude of resistance change increased with the increasing CO${}_{2}$ concentration. The response was calculated from the ratio between the average resistance during CO${}_{2}$ exposure and baseline resistance for which the drift component was taken into account.

**Figure 8:**
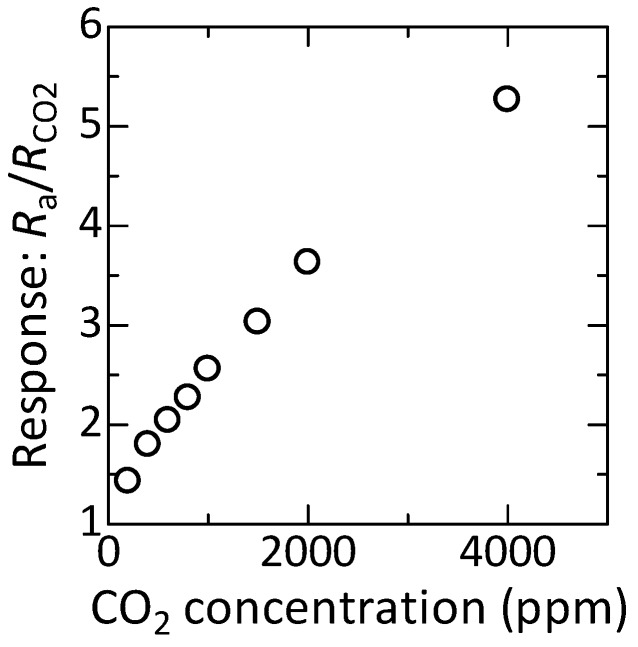
CO${}_{2}$ concentration dependence of the response at 400 °C.

The obtained response was plotted against CO${}_{2}$ concentration, as shown in Figure 8. In the measured concentration range, the response increased with CO${}_{2}$ concentration without saturation. Furthermore, it was demonstrated that a minimum amount of 200 ppm of CO${}_{2}$ was distinguishable in this study. Note that 200 ppm was the least measurement step in this study, and thus the device in this study has the potential of a better resolution, less than 200 ppm. Consequently, these results, including the discussion in Section 3.3, indicate the promising properties of the device in this study for smart CO${}_{2}$ sensors.

## 4. Conclusions

In this study, MHP-based CO${}_{2}$ sensors for smart sensors were characterized. The sensor comprised a La${}_{2}$O${}_{3}$/SnO${}_{2}$ stacked layer with Pt interdigitated electrodes, fabricated on a bridge-type MHP. SnO${}_{2}$ grains with a size of 20–40 nm formed a compact layer, whereas the La${}_{2}$O${}_{3}$ layer was discontinuously deposited on the SnO${}_{2}$ layer. The fabricated sensor showed a response of 2.9 for 1000-ppm CO${}_{2}$, which was relatively high compared with previously reported values. The resistance near the electrode contacts was suggested to significantly contribute to the response, causing a high response. Furthermore, CO${}_{2}$ detection was successfully performed in the range of 200–4000 ppm, and it was revealed that a minimum amount of 200-ppm CO${}_{2}$ was distinguishable by the sensor. The device in this study demonstrated promising properties for miniature low-power CO${}_{2}$ sensors toward smart sensing.

## Figures and Tables

**Table 1:** Comparison of the properties of conductmetric type CO${}_{2}$ sensors.

Material	Response for 1000-ppm CO${}_{2}$	Temperature	Ref.
La${}_{2}$O${}_{3}$-SnO${}_{2}$	1.5 ${}^{a}$	400 °C	[9]
LaOCl-SnO${}_{2}$ nanowire	4.5	400 °C	[14]
BaTiO-CuO	1.12	300 °C	[13]
Carbon nanotube	1.022 ${}^{b}$	RT	[16]
Graphene oxide	1.14	RT	[15]
PEI-PANI	1.08	RT	[17]

${}^{a}$: for approximately 1100-ppm CO${}_{2}$; ${}^{b}$: for 800 ppm CO${}_{2}$.

**Table 2:** Comparison of the properties of La-compound-based CO${}_{2}$ sensors.

Material	Response for 1000-ppm CO${}_{2}$	Temperature	MHP	Ref.
La${}_{2}$O${}_{3}$-SnO${}_{2}$	1.5 ${}^{a}$	400 °C	−	[9]
La${}_{2}$O${}_{3}$-SnO${}_{2}$	1.2	400 °C	−	[10]
LaOCl	2.9	260 °C	−	[35]
LaOCl-SnO${}_{2}$ NW	4.5	400 °C	−	[14]
La${}_{2}$O${}_{3}$/SnO${}_{2}$	2.9	350 °C	+	This work

${}^{a}$: for approximately 1100-ppm CO${}_{2}$.
